# Assessment of Spanish Translation of Websites at Top-Ranked US Hospitals

**DOI:** 10.1001/jamanetworkopen.2020.37196

**Published:** 2021-02-11

**Authors:** Christopher Cole Honeycutt, Karina Moreno Bueno, Tamara Tran, Michael Gao, Suresh Balu, Mark Sendak

**Affiliations:** 1 Trinity College of Arts and Sciences, Duke University, Durham, North Carolina; 2 Duke Institute for Health Innovation, Duke University School of Medicine, Durham, North Carolina

## Abstract

This cross-sectional study examines whether Spanish translation of top US hospital websites is associated with hospitals with public medical schools, children’s hospitals, larger Latinx population, or local Immigration and Customs Enforcement activities.

## Introduction

Coronavirus disease 2019 (COVID-19) has disproportionately affected Latinx communities across the United States. Latinx and African American individuals have 3 times the infection rate and twice the mortality rate from COVID-19 compared with White individuals.^1^ Latinx individuals are overrepresented as essential workers, which has contributed to their increased risk of contracting COVID-19 and experiencing related complications.^2^ Additional factors likely contribute to poor Latinx health outcomes, including institutional racism, discrimination, health care access, and socioeconomic and documentation status.^3^ As of 2019, the US population is 16.9% Latinx, of which 16.1% speak Spanish and do not speak English well.^4^ Many hospitals have implemented programs to improve accessibility of services to diverse populations. However, translation continues to be inadequate in addressing language barriers preventing Latinx communities from seeking appropriate care.^5^ Particularly, patients are deprived of important information about hospitals due to a paucity of Spanish translation of hospital websites. Our cross-sectional study aims to elucidate this issue by investigating potential variables associated with Spanish translation of hospital websites.

## Methods

This cross-sectional study did not involve human participants and, therefore, did not require ethical review under 45 CFR part 46. This study followed the Strengthening the Reporting of Observational Studies in Epidemiology (STROBE) reporting guideline.

A list of 30 top adult hospitals and 10 top children’s hospitals in the US was compiled from the *US News Weekly & World Report* and *Newsweek*. This list represents a snapshot of US health care, which consists of 637 total health systems, of which 54 contain at least one children’s hospital.^6^ Hospitals were classified as affiliated or not affiliated with a public medical school. Hospital websites were reviewed between June 29 and August 3, 2020. Websites were assessed for containing policies on Immigration and Customs Enforcement (ICE) activities on hospital grounds. The dependent variable was whether or not hospital websites had real-time Spanish translation capabilities, further categorized as using Google Translate or other translation methods. The 2018 American Community Survey Demographic and Housing Estimates were used to determine percentage of Latinx population in hospital counties. A threshold of *P* < .05 in logistic regression was used to determine statistical significance of variables on the binary outcome of a hospital website being available in Spanish. Odds ratios were calculated by exponentiating the coefficients of the logistic regression. Statistical analysis was performed using R statistical software version 1.2.5033 (R Project for Statistical Computing) from July to September 2020.

## Results

Of the 40 hospitals studied, nine (22.5%) had Spanish translation capabilities. Of those, 2 used Google Translate, whereas translation methods of the others are unknown. No hospital had explicit documentation of human translation of the website into Spanish. Table 1 displays analysis of the variables, including percentage of total hospitals, logistic regression coefficient, and odds ratio. Hospitals with public information about ICE enforcement were significantly more likely to have websites with Spanish translation (logistic regression coefficient, 2.41; odds ratio = 11.16 [95% CI, 1.30 to 127.13]; *P* = .03). Hospitals affiliated with a public medical school, children’s hospitals, and hospitals with a higher percentage of local Latinx population were not more likely to have websites with Spanish translation. Table 2 includes hospital names and raw data collected in this analysis.

**Table 1.** Likelihood of Spanish Translation of Hospital Websites

Variable	Proportion of total hospitals, % (No.)	Logistic regression coefficient	OR (95% CI)
Affiliated with public medical school	25 (10)	−1.79	0.17 (0.007 to 1.47)
Children’s hospital	25 (10)	1.54	4.68 (0.64 to 38.13)
Provides ICE information	15 (6)	2.41	11.16 (1.30 to 127.73)
Higher % Latinx population	NA	0.02	1.02 (0.97 to 1.08)

Abbreviations: ICE, Immigration and Customs Enforcement; NA, not available; OR, odds ratio.

**Table 2.** Top-Ranked US Hospital Characteristics and Website Spanish Translation

Hospital name	Public medical school	Latinx population, %	Children's hospital	ICE information	Website Spanish translation
Mayo Clinic-Rochester	No	6.1	No	No	Yes
Massachusetts General Hospital	No	20.0	No	No	Yes
Johns Hopkins Hospital	No	5.5	No	Yes	Yes
Cleveland Clinic	No	12.3	No	No	No
New York-Presbyterian Hospital-Columbia and Cornell	No	29.2	No	No	No
UCLA Medical Center	Yes	48.9	No	Yes	Yes
USCF Medical Center	Yes	15.2	No	Yes	No
Cedars-Sinai Medical	No	48.9	No	No	No
NYU Langone Hospitals	No	29.2	No	No	No
Northwestern Memorial Hospital	No	28.7	No	No	No
University of Michigan Hospitals-Michigan Medicine	Yes	4.5	No	No	No
Stanford Health Care-Stanford Hospital	No	14.7	No	No	No
Brigham and Women's Hospital	No	20.0	No	Yes	Yes
Mount Sinai Hospital	No	29.2	No	No	No
UPMC Presbyterian Shadyside	Yes	3.4	No	No	No
Keck Hospital of USC	No	48.9	No	No	No
University of Wisconsin Hospitals	Yes	7.3	No	No	No
Hospitals of the University of Pennsylvania-Penn Presbyterian	No	15.2	No	No	No
Mayo Clinic-Phoenix	No	43.0	No	No	Yes
Houston Methodist Hospital	No	44.9	No	No	No
Yale New Haven Hospital	No	27.2	No	No	No
Duke University Medical Center	No	14.3	No	No	No
Boston's Children's Hospital	No	19.7	Yes	No	Yes
Children's Hospital of Philadelphia	No	14.5	Yes	No	No
Cincinnati Children's Hospital Medical Center	Yes	3.7	Yes	No	No
Texas Children's Hospital	No	44.8	Yes	No	No
Children's Hospital Los Angeles	No	48.6	Yes	No	Yes
Children's Hospital Colorado	Yes	28.4	Yes	No	No
Children's National Hospital	No	10.9	Yes	No	Yes
Nationwide Children's Hospital	Yes	5.9	Yes	No	No
UPMC Children's Hospital of Pittsburgh	Yes	3.1	Yes	No	No
Lucile Packard Children's Hospital Stanford	No	5.7	Yes	No	No
University of Colorado Hospital	Yes	29.2	No	No	No
University of Washington Medical Center	No	7.3	No	Yes	No
Emory University Hospital	No	4.6	No	No	No
University Hospitals Cleveland Medical Center	No	12.3	No	No	No
Rush University Medical Center	No	28.7	No	No	No
Barnes Jewish Hospital	No	3	No	No	No
Vanderbilt University Medical Center	No	1.7	No	No	No
Scripps Memorial Hospital La Jolla	No	34	No	Yes	No
Total counts, No.	10	20.57	10	6	9

Abbreviation: ICE, Immigration and Customs Enforcement.

## Discussion

We found that only 22.5% of top hospitals in the United States have websites with Spanish translation. This study highlights the substantial and persistent language barriers that Latinx communities face when accessing health care at hospitals. We found that ICE enforcement information was significantly associated with Spanish translation of hospital websites. Location in a county with more Latinx residents was not associated with Spanish translation.

We acknowledge substantial limitations of this study, including the small sample size and small number of analyzed variables. In the era of COVID-19, when health disparities based on race, ethnicity, and socioeconomic status have come into greater focus, it is critical for hospitals to provide equitable access to care to their entire patient populations. This can begin with the simple act of translating websites.
