# Error in End Matter

**DOI:** 10.1001/jamanetworkopen.2022.6227

**Published:** 2022-03-22

In the Research Letter titled “Assessment of Spanish Translation of Websites at Top-Ranked US Hospitals,”^1^ published February 11, 2021, there was an error in a co-author’s last name in the Author Affiliations of the Article Information section. The last name should have appeared as Moreno Bueno. This article has been corrected.^1^
